# Infection history of the blood-meal host dictates pathogenic potential of the Lyme disease spirochete within the feeding tick vector

**DOI:** 10.1371/journal.ppat.1006959

**Published:** 2018-04-05

**Authors:** Bharti Bhatia, Chad Hillman, Valentina Carracoi, Britney N. Cheff, Kit Tilly, Patricia A. Rosa

**Affiliations:** Laboratory of Bacteriology, Rocky Mountain Laboratories, Division of Intramural Research, National Institute of Allergy and Infectious Diseases, National Institutes of Health, Hamilton, MT United States of America; Medical College of Wisconsin, UNITED STATES

## Abstract

Lyme disease in humans is caused by several genospecies of the *Borrelia burgdorferi* sensu lato (s.l.) complex of spirochetal bacteria, including *B*. *burgdorferi*, *B*. *afzelii* and *B*. *garinii*. These bacteria exist in nature as obligate parasites in an enzootic cycle between small vertebrate hosts and Ixodid tick vectors, with humans representing incidental hosts. During the natural enzootic cycle, infected ticks in endemic areas feed not only upon naïve hosts, but also upon seropositive infected hosts. In the current study, we considered this environmental parameter and assessed the impact of the immune status of the blood-meal host on the phenotype of the Lyme disease spirochete within the tick vector. We found that blood from a seropositive host profoundly attenuates the infectivity (>10^4^ fold) of homologous spirochetes within the tick vector without killing them. This dramatic neutralization of vector-borne spirochetes was not observed, however, when ticks and blood-meal hosts carried heterologous *B*. *burgdorferi* s.l. strains, or when mice lacking humoral immunity replaced wild-type mice as blood-meal hosts in similar experiments. Mechanistically, serum-mediated neutralization does not block induction of host-adapted OspC+ spirochetes during tick feeding, nor require tick midgut components. Significantly, this study demonstrates that strain-specific antibodies elicited by *B*. *burgdorferi* s.l. infection neutralize homologous bacteria within feeding ticks, before the Lyme disease spirochetes enter a host. The blood meal ingested from an infected host thereby prevents super-infection by homologous spirochetes, while facilitating transmission of heterologous *B*. *burgdorferi* s.l. strains. This finding suggests that Lyme disease spirochete diversity is stably maintained within endemic populations in local geographic regions through frequency-dependent selection of rare alleles of dominant polymorphic surface antigens.

## Author summary

Lyme disease is a tick-borne infection of humans that is caused by a spirochetal bacterium called *Borrelia burgdorferi*. It is a zoonosis, which means that these bacteria exist in nature outside of people. Many different strains of *B*. *burgdorferi* are stably maintained in the same local population of infected wild animals and ticks. Once infected, people and animals are immune to re-infection by the same strain, but can become infected with a different *B*. *burgdorferi* strain. We previously assumed that factors in the blood of an immune host recognized closely related bacteria and neutralized them after they were transmitted by a feeding tick. However, in the current study we found that this actually occurs within feeding ticks, even before the Lyme disease spirochete enters the immune host. Conversely, when ticks and animals are infected with different strains of *B*. *burgdorferi*, blood ingested by feeding ticks enhances the spirochete’s ability to re-infect the host. These findings provide insight into the evolutionary processes that shape the natural diversity of the Lyme disease spirochete and expose a highly vulnerable stage within the tick vector for targeting effective vaccines. They also identify a way to test whether a potential Lyme disease vaccine would protect against tick-borne spirochetes.

## Introduction

*Borrelia burgdorferi* sensu lato (s.l.), the spirochetal agents of Lyme disease, comprise several closely related genospecies of pathogenic bacteria that are maintained in nature in an enzootic cycle involving *Ixodes* ticks and a wide range of vertebrate hosts. While these spirochetes can cause Lyme disease in humans, most natural reservoir hosts become persistently infected without signs of disease [1–5]. *Ixodes* ticks feed once per life stage and the corresponding blood meal is required for the immature larval and nymphal stages to molt, and for the adult females to lay eggs. Acquisition of *B*. *burgdorferi* s.l. typically occurs when larval ticks feed on an infected host. Ingested spirochetes colonize the larval tick midgut, survive through the molt and are subsequently transmitted to new vertebrate hosts by feeding nymphs. Persistent infection of these vertebrate hosts and subsequent acquisition of *B*. *burgdorferi* by feeding larval ticks complete the infectious cycle.

Ingestion of host blood by infected ticks stimulates spirochete replication and induces changes that are critical for transmission of *B*. *burgdorferi* s.l. to the vertebrate host and survival in this disparate environment [6–10]. In contrast to the highly infectious phenotype of spirochetes in replete ticks, a recent study from our lab demonstrated that spirochetes colonizing unfed ticks are viable, but essentially non-infectious [11]. We will use the term “pathogenic potential” rather than “virulence”, as proposed by Casadevall [12], to describe the infectious phenotype of wild-type *B*. *burgdorferi* s.l. in an experimental mouse-tick cycle because infection does not cause disease in these rodent hosts. We conclude that in addition to stimulating spirochete replication, exposure to vertebrate blood during tick feeding also induces phenotypic changes that conditionally prime *B*. *bugdorferi* s.l. for subsequent infection of a vertebrate host, thereby dramatically enhancing the pathogenic potential of tick-borne spirochetes.

In nature, multiple strains of *B*. *burgdorferi* s.l. are stably maintained at high prevalence in both the tick vector and reservoir hosts sharing the same local geographic area [13–20]. Such diversity is observed within the single genospecies of Lyme disease spirochete, *B*. *burgdorferi s*.s, that predominates in North America, and among strains of the three genospecies, *B*. *burgdorferi*, *B*. *afzelii and B*. *garinii*, that co-exist and cause Lyme disease in Eurasia. Laboratory and field studies indicate that infection of vertebrates with *B*. *burgdorferi* s.l. elicits strain-specific protective immunity [21–30]. In an endemic region, infected nymphal ticks will occasionally feed on infected hosts carrying the same (homologous) or different (heterologous) *B*. *burgdorferi* s.l. strains. In the current study, we have extended our previous observation of conditional priming of *B*. *burgdorferi* s.l. during tick feeding to investigate how host immune status impacts the pathogenic potential of spirochetes within infected ticks. We show that blood from an infected host can have either a profoundly negative or positive impact on the infectious phenotype of the Lyme disease spirochete within feeding ticks, depending upon the similarity of the strains. This dichotomous response to host blood prevents super-infection by the homologous *B*. *burgdorferi* s.l. strain, while promoting infection by heterologous strains. Significantly, this study demonstrates for the first time that protective immunity against the Lyme disease spirochete, like induction of pathogenic potential, takes effect within the feeding tick vector prior to transmission, through a neutralization mechanism that does not require bacterial killing nor utilize tick midgut components.

## Results

### Experimental approach

**Fig 1:**
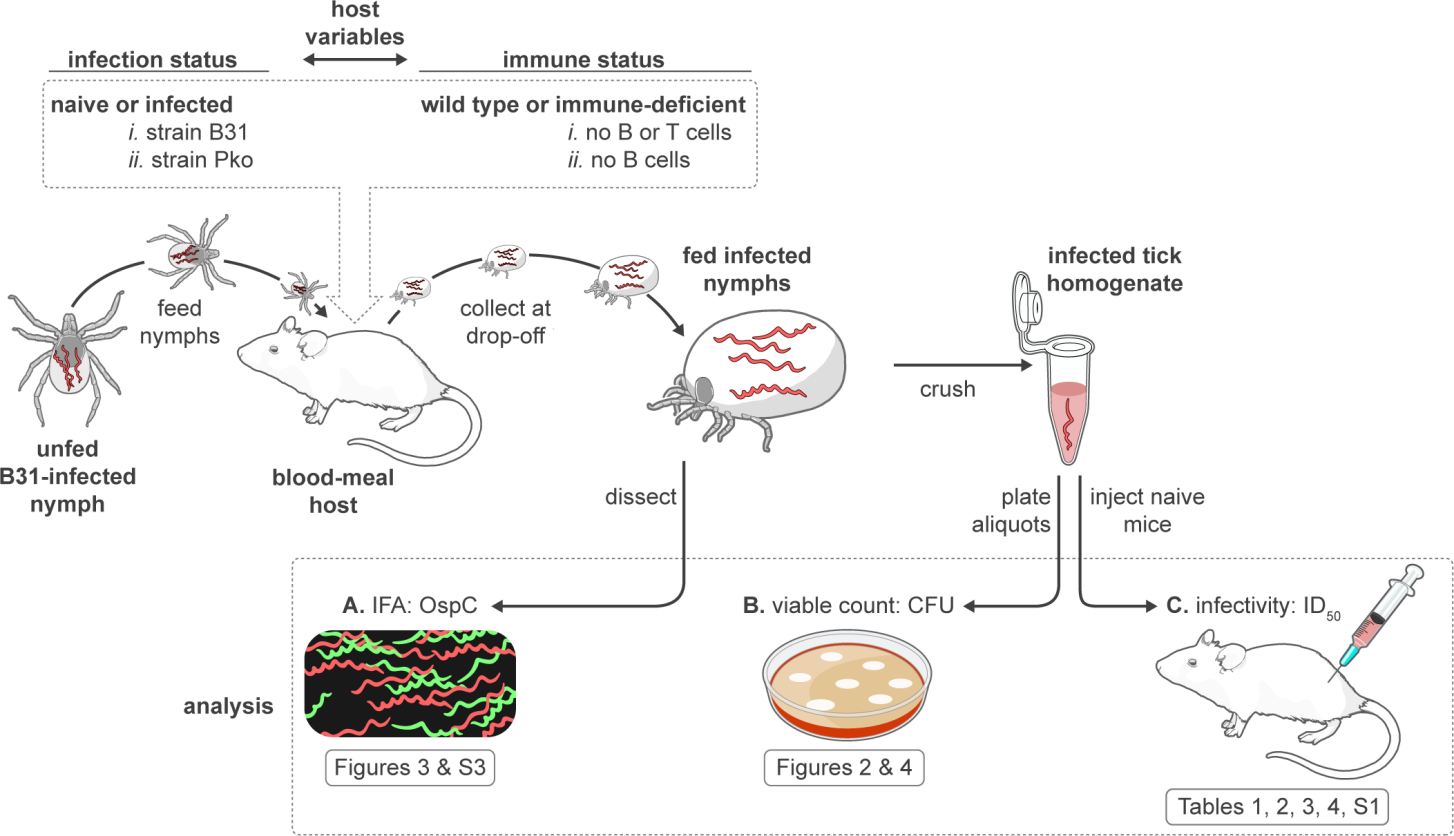
Experimental assessment of infectious phenotype of spirochetes in fed nymphs. *Ixodes scapularis* nymphs (infected as larvae with *B*. *burgdorferi* strain B31) were fed on naïve mice, on mice infected with the homologous strain (B31), or on mice infected with a heterologous strain (PKo) and collected after feeding to repletion. Blood-meal hosts representing different strains of wild-type and immune-deficient laboratory mice were used in separate experiments. **A.)** OspC+ spirochetes in the midguts of a subset of fed ticks from each mouse/tick cohort were visualized by immunofluorescence assay (IFA) with a monoclonal antibody specific for OspC and a polyclonal anti-*B*. *burgdorferi* serum to counter-stain midgut spirochetes **B.)** Viable spirochetes in homogenates prepared from the remaining fed ticks (pooled) in each mouse/tick cohort were quantified by plating an aliquot for colony forming units (CFU). **C.)** Mice were injected with serial dilutions containing defined numbers of viable spirochetes from fed tick homogenates to assess the relative infectivity of each experimental group.

We assessed the impact of the immune status of the blood-meal host on the infectious phenotype of the Lyme disease spirochete within the tick vector, as schematically diagrammed in Fig 1 and outlined as follows. Groups of infected nymphs were fed to repletion on naïve mice, on mice infected with the homologous *B*. *burgdorferi* strain (B31), or on mice infected with a heterologous *B*. *afzelii* strain (PKo). Wild-type (WT) and immune-deficient laboratory mice were utilized in separate experiments. Fed nymphs were collected at drop-off and the presence of OspC+ spirochetes in the tick midgut was analyzed by IFA using several ticks from each experimental group. The remaining fed nymphs were pooled per individual mouse and crushed to yield infected tick homogenates. Aliquots of these homogenates were plated to quantify viable spirochetes and thereby calculate the average spirochete load per fed tick in each pool and inocula. Serial dilutions of fed tick homogenates containing defined numbers of viable spirochetes were injected into naïve WT mice to assess the impact of the host blood meal / immunity on the pathogenic potential of spirochetes within infected ticks.

### Modest impact of host immunity on spirochete burden in fed ticks

**Fig 2:**
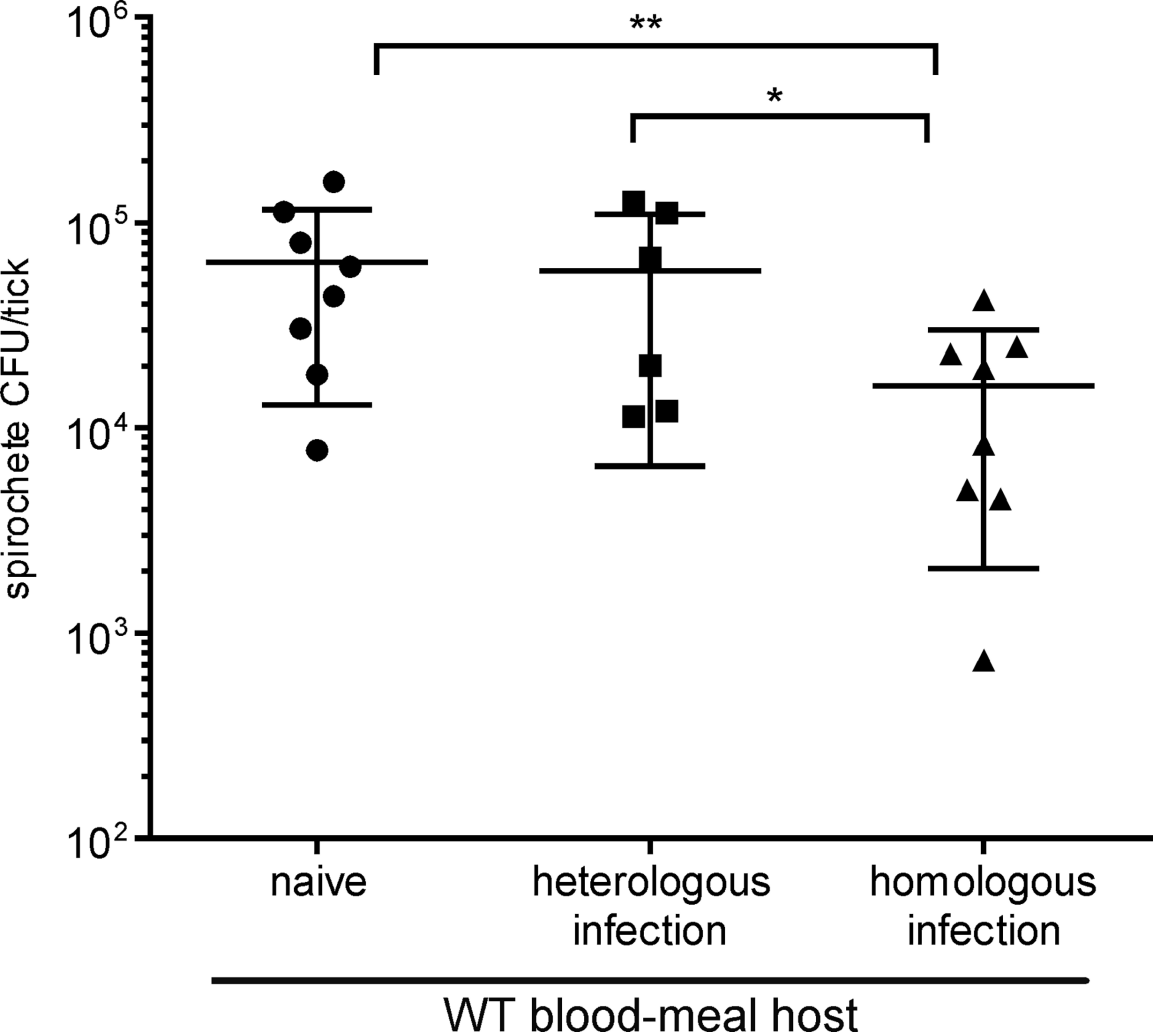
Spirochete burden in infected nymphs fed on naïve and infected wild-type mice. Cohorts of nymphs infected as larvae with *B*. *burgdorferi* strain B31 were fed to repletion on groups of naïve, heterologously-infected (PKo) or homologously-infected (B31) mice, as identified at the bottom of the graph. Fed nymphs were collected at drop-off, pooled for individual mouse/tick cohorts, and crushed. The number of viable spirochetes per tick was estimated by plating an aliquot of each pooled homogenate for colony forming units (CFU). Each point on the graph represents the average spirochete load per tick for each cohort of 5–12 infected nymphs fed upon individual mice, with a total of 6–8 animals per group. (**)P = 0.02 naïve versus homologous; (*)P = 0.1 heterologous versus homologous; P = 0.8 naïve versus heterologous; P values calculated using non-parametric rank order test.

Ingestion of host blood promotes spirochete replication in infected nymphs [31]. To determine whether the acquired immune response of an infected blood-meal host impacts spirochete replication in the tick midgut, we quantified the spirochete burden in infected nymphs after they fed to repletion on naïve, homologously infected, or heterologously infected wild-type (WT) mice. The results from four independent experiments were combined and the mean number of viable spirochetes per tick in each mouse/tick cohort was estimated (Fig 2). The spirochete burdens in infected ticks fed on naïve or heterologously infected mice were similar (6.4×10^4^ vs 5.8×10^4^, respectively), and approximately 4-fold greater than the spirochete burden in the same cohort of infected ticks fed on mice infected with the homologous strain (1.6×10^4^). These results demonstrate a modest strain-specific impact on spirochete burden when infected nymphs feed upon immune hosts.

### Severely limited pathogenic potential of spirochetes in ticks fed on homologously infected wild-type mice

In addition to fostering replication, spirochetes in the tick midgut undergo biological changes in response to ingested blood that enable their subsequent transmission and infection of the vertebrate host [7–11, 32]. Therefore, we compared the infectious dose of spirochetes derived from infected ticks fed on naive versus immune hosts to measure their relative pathogenic potentials. Fed tick homogenates containing known numbers of viable organisms (as outlined in Fig 1 and described above) were used to needle-inoculate naive mice with defined doses ranging from 10−10^5^ spirochetes per mouse, using 3 to 6 mice per dose. *B*. *burgdorferi* infection in mice was determined by seroconversion and isolation of spirochetes from tissues; the data from several experiments were combined and are presented in Table 1.

**Table 1:** Infectivity of spirochetes in infected nymphs after feeding on naïve or infected WT mice.

infection status of blood-meal host^1^	number of spirochetes in inoculum^2^	number of mice infected/total naïve mice injected^3^	total infected mice per group^4^
naïve	10	2/3	**37/39**
	10^2^	8/8	
	10^3^	13/14	
	6x10^3^	6/6	
	10^4^	3/3	
	10^5^	5/5	
heterologous	10	3/3	**22/24**
	10^2^	3/3	
	10^3^	7/9	
	6x10^3^	6/6	
	10^4^	3/3	
homologous	10^2^	0/3	6/39^*^
	10^3^	1/9	
	6x10^3^	0/6	
	10^4^	1/8	
	10^5^	4/13	

^1^ Cohorts of nymphs infected as larvae with *B*. *burgdorferi* strain B31 were fed to repletion on groups of naïve, heterologously-infected (PKo) or homologously-infected (B31) RML mice (WT blood-meal host).

^2^ Homogenates prepared from pools of fed nymphs were used to needle-inoculate WT naïve mice with doses ranging from 10−10^5^ spirochetes per mouse, as enumerated and confirmed by plating.

^3^ *B*. *burgdorferi* infection in mice was determined by seroconversion to whole cell lysates by immunoblot analysis as shown in S1 Fig, and isolation from ear, bladder and joint tissues; infected mice were positive by all measures. Data shown are the combined results of three separate experiments.

^4^ P values calculated using Fisher’s exact test for infectious outcomes with different blood-meal hosts.

* P = 2.69e-13, for homologous versus naïve; P = 1.45e-9 for homologous versus heterologous; P = 0.63 for heterologous versus naive blood-meal hosts.

Spirochetes in homogenates prepared from infected nymphs were highly infectious after ticks fed on naïve mice, or after feeding on mice infected with the heterologous strain: 5/6 mice became infected following injection of ~10 organisms, and 59/63 mice were infected with doses ranging up to 10^5^ organisms from these sources (Table 1). In contrast, the infectivity of viable spirochetes in infected nymphs was dramtically reduced after ticks fed on mice infected with the homologous strain, where only 6/29 mice were infected with doses ranging from 10^2^ to 10^5^ organisms (Table 1). This outcome indicates an approximately 10^4^-fold difference in the infectious dose of viable spirochetes derived from ticks fed on homologously infected hosts (≥ 10^5^ organisms) versus naïve or heterologously infected hosts (≤ 10 organisms). These results demonstrate that the immune status and infection history of the vertebrate host on which infected nymphs feed will strongly impact the pathogenic potential of the viable spirochetes they can transmit.

### Homologous host immunity does not prevent or ablate OspC^+^ spirochetes in nymphs

**Fig 3:**
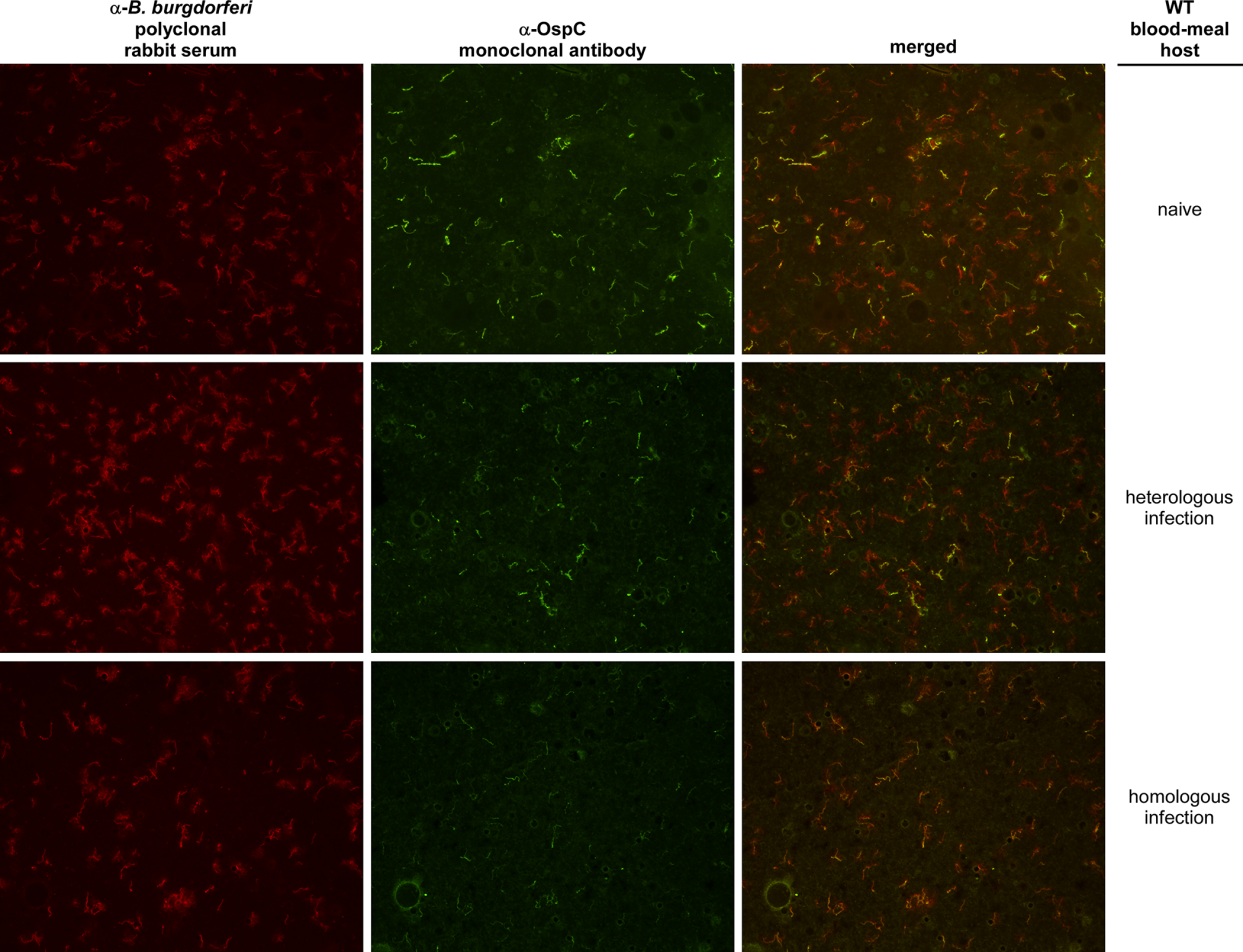
OspC production by spirochetes in infected nymphs fed on naïve or infected wild-type mice. Dissected midguts of strain B31-infected nymphs fed on naïve, heterologously infected (strain PKo) or homologously infected (strain B31) mice, as identified to the right of the images, were co-stained with a rabbit polyclonal anti-*B*. *burgdorferi* serum and a mouse monoclonal antibody that recognizes OspC. Primary antibody binding, as identified above the panels, was visualized on a fluorescent microscope (20X magnification) with TRITC- (total *B*. *burgdorferi*) and FITC- (OspC+ *B*. *burgdorferi*) tagged secondary antibodies. The entire experiment and IFA analysis were conducted 4 times, with visual assessment of 6 nymphs per group, and 5 fields per nymph. Representative IFA images are shown.

OspC is an essential outer surface lipoprotein of *B*. *burgdorferi* that is required for spirochete survival at the initial stage of mammalian infection [32, 33]. Expression of *ospC* is induced by environmental cues that spirochetes encounter in the midgut of feeding ticks and is a hallmark of a global adaptive response that prepares *B*. *burgdorferi* for host infection [7, 34–36]. Therefore, as a measure of the host-adaptive response, we also assessed whether OspC was present on spirochetes in ticks fed on immune hosts. A subset of infected nymphs fed on naïve or infected WT mice were subjected to analysis by IFA, using a polyclonal anti-*B*. *burgdorferi* antiserum to visualize the spirochete population in dissected tick midguts, and a monoclonal antibody to identify the subset of host-adapted spirochetes producing OspC (Fig 3). Negative control images of an uninfected tick midgut, or infected midguts without primary or secondary antibodies, demonstrate specificity of antibody staining relative to background autofluorescence (S2 Fig). All groups of infected ticks contained OspC+ spirochetes (Fig 3), irrespective of the naïve or infected status of their blood-meal hosts or stark differences in the infectious phenotypes of these spirochetes (Table 1). These data, coupled with the data in Fig 2 and Table 1, demonstrate that the blood meal from an infected host neutralizes homologous spirochetes in the infected tick midgut without killing them, blocking their host-adaptive response, or eliminating OspC+ organisms.

### Full pathogenic potential of spirochetes in ticks fed on immune-deficient mice

**Fig 4:**
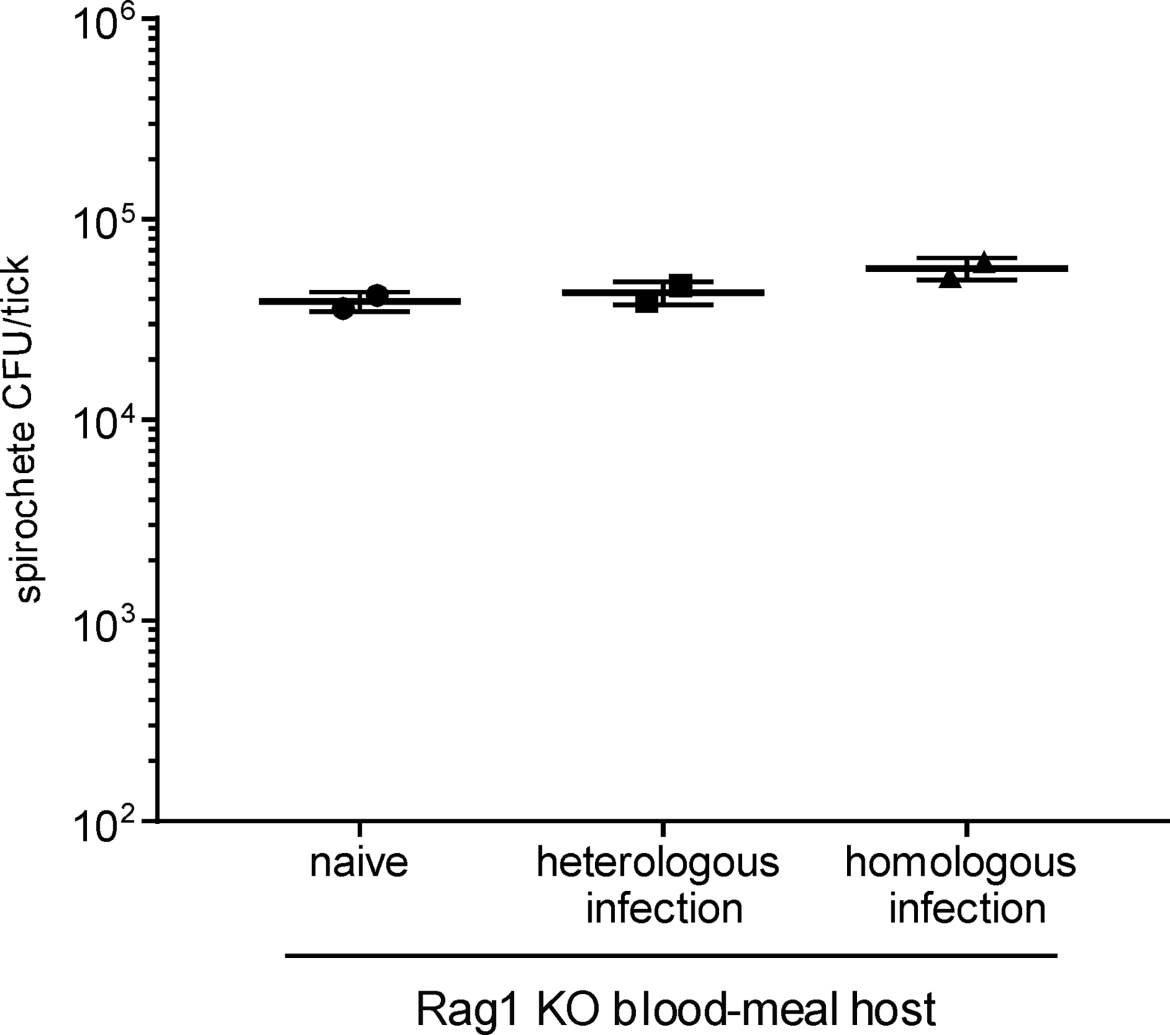
Spirochete burden in infected nymphs fed on naïve and infected Rag1 KO mice. Cohorts of nymphs infected as larvae with *B*. *burgdorferi* strain B31 were fed to repletion on groups of naïve, heterologously infected (PKo) or homologously infected (B31) Rag1 KO mice, as identified at the bottom of the graph. Fed nymphs were collected at drop-off, pooled for individual mouse/tick cohorts, and crushed. The number of viable spirochetes per tick was estimated by plating an aliquot of each pooled homogenate for colony forming units (CFU). Each point on the graph represents the average spirochete load per tick for each cohort of 5–12 infected nymphs fed upon individual mice, with a total of 2 animals per group. The number of viable spirochetes per tick was estimated by plating an aliquot of the pooled homogenate of crushed ticks from each mouse/tick cohort for colony forming units (CFU). Naïve versus homologous P = 0.3; heterologous versus homologous P = 0.3; naïve versus heterologous P = 0.6; calculated using non-parametric rank order test.

We next assessed whether the acquired immune response of infected mice was responsible for the observed neutralization of homologous spirochetes in fed ticks. Rag1 KO mice are incapable of mounting an adaptive immune response due to a lack of functional B and T lymphocytes. Hence, blood meals from both naïve and infected Rag1 KO mice lack antibodies to *B*. *burgdorferi*. Similar to previous experiments, we quantified the mean number of viable spirochetes per tick in cohorts of infected nymphs fed upon naïve, heterologously and homologously infected Rag1 KO mice and found that the infection status of the blood-meal host did not affect the spirochete burden in any group of ticks (~ 3–5 x10^4^ CFU/tick) (Fig 4). We then inoculated naïve wild-type mice with defined doses of spirochetes derived from infected nymphs fed on Rag1 KO mice, as in previous experiments, and monitored infection in recipient mice. No difference in infectivity was observed among spirochetes from ticks fed on naïve, heterologously or homologously infected Rag1 KO mice (Table 2). Some mice became infected with as few as 10 organisms from each source, and all mice (5/5 per group) were infected with doses of 10^3^ organisms or higher, regardless of the infectious status of the blood-meal host. This outcome contrasts sharply with what we previously observed with spirochetes in ticks fed on immune-competent WT mice (Table 1).

**Table 2:** Infectivity of spirochetes in infected nymphs fed on naïve and infected Rag1 KO mice.

infection status of blood-meal host^1^	number of spirochetes in inoculum^2^	number of mice infected/total naïve mice injected^3^	total infected mice per group^4^
naïve	5	1/5	**13/18**
	50	4/5	
	5x10^2^	5/5	
	5x10^3^	3/3	
heterologous	10	5/5	**18/18**
	10^2^	5/5	
	10^3^	5/5	
	10^4^	3/3	
homologous	10	2/5	**14/18**
	10^2^	4/5	
	10^3^	5/5	
	10^4^	3/3	

^1^ Cohorts of nymphs infected as larvae with *B*. *burgdorferi* strain B31 were fed to repletion on groups of naïve, heterologously-infected (PKo) or homologously-infected (B31) Rag1 KO mice (blood-meal host).

^2^ Homogenates prepared from pools of fed ticks were used to needle-inoculate naïve wild-type mice with the indicated number of viable organisms, as enumerated and confirmed by plating

^3^ *B*. *burgdorferi* infection in WT mice inoculated with fed tick homogenates was determined by seroconversion to whole cell lysates by immunoblot analysis, and isolation from ear, bladder and joint tissues; infected mice were positive for all measures.

^4^ P = 1 for all comparisons (homologous versus naïve; homologous versus heterologous; heterologous versus naive blood-meal hosts). P values calculated using Fisher’s exact test for infectious outcomes with different blood-meal hosts.

To confirm that this outcome stemmed from the immune-deficient status of the Rag1 KO blood-meal host, and not differences in the genetic backgrounds of strains (inbred C57Bl/6, “B6” versus outbred Swiss-Webster, “RML”), we conducted a similar experiment using immune-competent WT B6 mice. As observed previously with immune-competent RML mice, the infectious phenotype of viable spirochetes in ticks fed on homologously infected WT B6 mice was severely attenuated (1/18 mice infected with doses ranging from 10 to 10^4^ tick-derived spirochetes), whereas most mice were infected (32/36) with similar doses of spirochetes derived from ticks fed on naive or heterologously infected B6 mice (S1 Table).

We next performed a similar experiment using immune-deficient muMT^-^ mice (B6 background), which contain T cells, but lack mature antibody-producing B cells (Table 3). As with Rag1 KO mice, the infection history of the muMT^-^ blood-meal host had no impact on the pathogenic potential of spirochetes in fed ticks. Some mice were infected at the lowest dose of 10 spirochetes from all 3 sources, and all mice were infected with doses of 10^3^ and higher (Table 3). These results establish that antibodies, which are not present in blood ingested from muMT^-^ mice, comprise the strain-specific neutralizing component of homologously infected host blood seen in previous experiments with wild-type mice.

**Table 3:** Infectivity of spirochetes in infected nymphs fed on naïve and infected muMT^-^ mice.

infection status of blood-meal host^1^	number of spirochetes in inoculum^2^	number of mice infected/total naïve mice injected^3^	total infected mice per group^4^
naïve	10	1/5	**13/18**
	10^2^	4/5	
	10^3^	5/5	
	10^4^	3/3	
heterologous	10	2/5	**12/18**
	10^2^	2/5	
	10^3^	5/5	
	10^4^	3/3	
homologous	10	1/5	**13/18**
	10^2^	4/5	
	10^3^	5/5	
	10^4^	3/3	

^1^ Cohorts of nymphs infected as larvae with *B*. *burgdorferi* strain B31 were fed to repletion on groups of naïve, heterologously-infected (PKo) or homologously-infected (B31) muMT^-^ mice (blood-meal host).

^2^ Homogenates prepared from pools of fed ticks were used to needle-inoculate naïve wild-type mice with the indicated number of viable organisms, as enumerated and confirmed by plating

^3^ *B*. *burgdorferi* infection in WT mice inoculated with fed tick homogenates was determined by seroconversion to whole cell lysates by immunoblot analysis, and isolation from ear, bladder and fat tissues.

^4^ P = 1 for all comparisons (homologous versus naïve; homologous versus heterologous; heterologous versus naive blood-meal hosts). P values calculated using Fisher’s exact test for infectious outcomes with different blood-meal hosts.

### Neutralization of spirochete infectivity by incubation with homologous immune serum

The previous experiments identify strain-specific antibodies as the component of host blood that neutralizes spirochetes within the midguts of infected nymphs. However, we questioned whether some aspect of the tick midgut environment might also contribute to spirochete neutralization. To address this possibility, we utilized highly infectious homogenates prepared from infected nymphs fed upon naive mice (infectious dose of ~10 spirochetes, Table 1). We briefly exposed aliquots of this tick homogenate to an equal volume of serum from naive or infected mice for 30 minutes at room temperature. We also incubated an aliquot of the same infected tick homogenate with PBS as an untreated control. We then injected naive mice with the equivalent of 10^3^ organisms present in the original homogenate to assess the impact of serum treatment on spirochete infectivity and plated to determine the viability of spirochetes in the inoculum after incubation with serum or PBS. This ex vivo experiment fully reproduced the results of previous experiments with spirochetes derived from infected nymphs fed directly upon immune blood-meal hosts (Table 4). Spirochetes retained an infectious phenotype after incubation with PBS (5/5 mice infected) or exposure to sera from naive or heterologously infected mice (9/10 mice infected), whereas infectivity was ablated (0/5 mice infected) when the same tick homogenate was incubated with serum from an homologously infected host (Table 4). Plating confirmed that spirochete viability was not reduced by incubation with serum, irrespective of the immune status of the host from which they were obtained. This experiment demonstrates that strain-specific antibodies can directly attenuate the pathogenic potential of host-adapted spirochetes without impacting their viability or requiring exogenous tick factors. This brief exposure to serum does not allow sufficient time for a global adaptive response in gene expression and protein synthesis. Hence the mechanism of neutralization appears to be an immediate and direct consequence of antibody binding to the spirochetal outer surface.

**Table 4:** Infectivity of spirochetes in tick homogenates after exposure to immune serum.

treatment of B31-infected tick homogenate^1^	# of spirochetes injected^2^	# of mice infected/total naïve mice injected^3^
PBS	790	5/5
**naïve** serum	840	4/5
anti-PKo serum (**heterologous**)	890	5/5
anti-B31 serum (**homologous**)	860	0/5*

^1^ Aliquots of an homogenate prepared from B31-infected nymphs fed to repletion on naïve WT mice (as described and analyzed in Table 1) were individually incubated at room temperature for 30 minutes with equal volumes of immune or naive mouse serum, or PBS, as indicated.

^2^ Naïve WT mice were injected with a dose of ~10^3^ viable spirochetes (CFU) as determined for the original homogenate; the actual number of viable spirochetes injected was determined by plating the individual inocula prepared from treated homogenates.

^3^ *B*. *burgdorferi* infection in mice was determined by seroconversion to whole cell lysates by immunoblot analysis, and isolation from ear, bladder and fat tissues; infected mice were positive by all measures.

* P = 0.0079 for homologous (anti-B31) vs heterologous (anti-PKo) serum or PBS; P = 0.04 for homologous (anti-B31) vs naïve serum; P values calculated using Fisher’s exact test.

### Broad reactivity of infected mouse sera with homologous and heterologous strains

**Fig 5:**
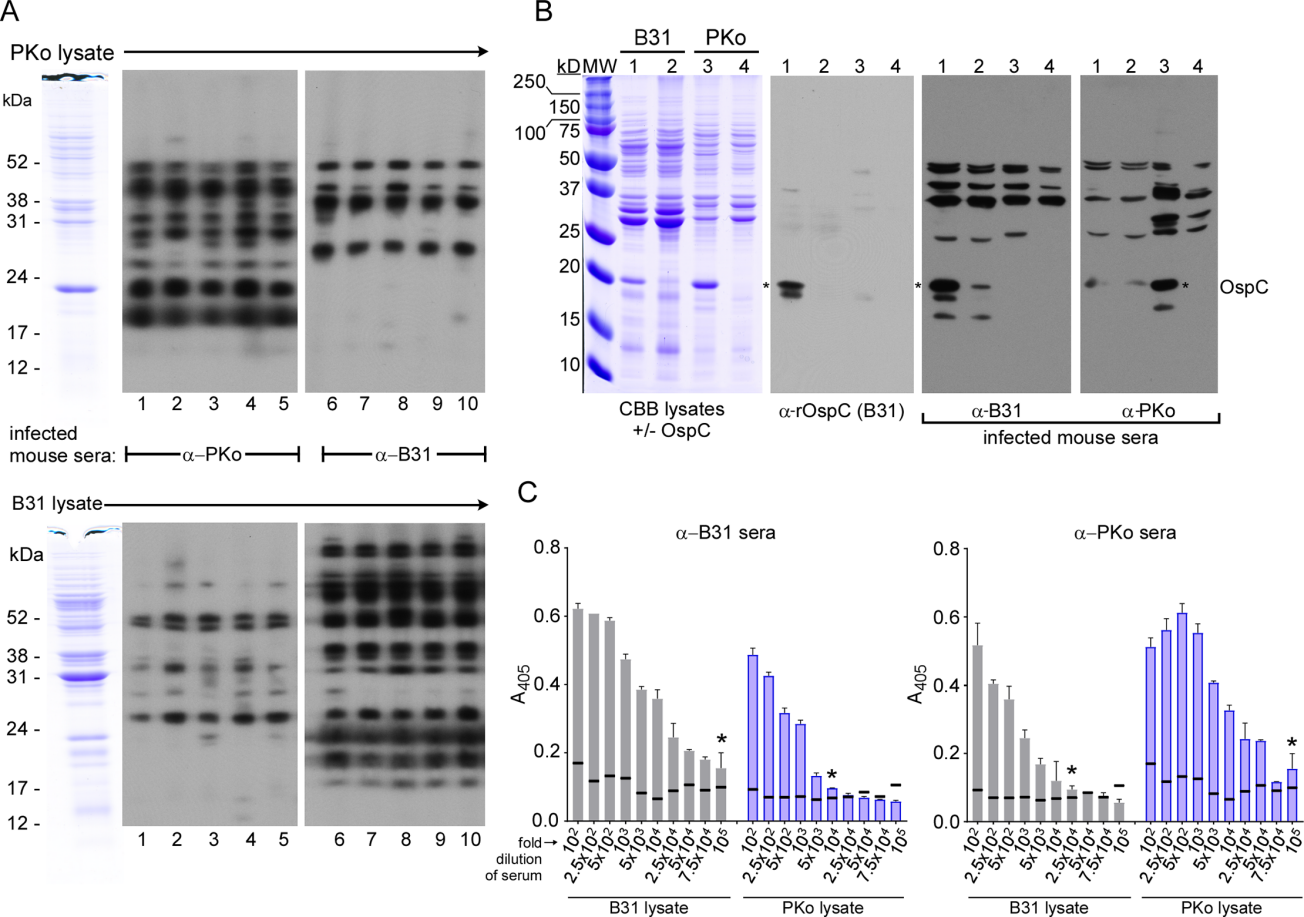
Serologic response of infected mice to homologous and heterologous *B*. *burgdorferi* strains. **A.)** Representative immunoblots with sera of mice infected with *B*. *afzelii* strain PKo (lanes 1–5) or *B*. *burgdorferi* strain B31 (lanes 6–10) against whole cell lysates of strains PKo (top panels) and B31 (bottom panels) **B.)** Whole cell lysates of strains B31 (lanes 1–2) and PKo (lanes 3–4), with or without OspC (lanes 1 & 3 versus 2 & 4, respectively), stained with coomassie brilliant blue (CBB) to visualize all proteins (left-most panel), or transferred to membranes and incubated with infected mouse sera (panels on the right side of the figure). A blot containing the same lysates was also incubated with polyclonal rabbit antiserum raised against recombinant OspC from strain B31 (second panel from the left). The mobility of OspC is indicated at the right side of the figure. Molecular weight markers (MW) are visible at the left side of the figures, with mass indicated (kD). **C.)** ELISA titers of pooled sera from 5 mice infected with either strain B31 or PKo and tested against lysates of homologous and heterologous strains, as identified beneath the graphs. Each bar represents the average of three technical replicates, with the standard deviation shown. Baseline absorbances were determined for the same dilutions of pooled pre-immune sera, in triplicate, against B31-S9 and PKo lysates. The threshold for positive sero-reactivity was set at 3 standard deviations above the mean absorbance of pre-immune sera at each dilution, indicated by a dark line. The ELISA titer represents the highest dilution of immune sera at which absorbance above this baseline cut-off was achieved, as indicated by asterisks.

We directly compared the antibody responses of B31- and PKo-infected wild-type mice by immunoblot analysis with whole cell lysates of both strains, including clonal derivatives of strains B31 and PKo that make or lack OspC (Fig 5). Antibodies in sera of mice infected with either strain recognized multiple proteins in both lysates, indicating a fairly broad and cross-reactive immune response accompanying infection, but with stronger recognition of more proteins in lysates from the homologous versus heterologous strain (Fig 5A, compare top and bottom panels). Significantly, OspC was only detected by homologous sera, indicating strict strain-specific recognition of this abundant surface protein by the polyclonal antibody response of B31- and PKo-infected hosts (Fig 5B). This result is consistent with previous reports [37–40] and indicates that antibodies recognizing OspC could contribute to the observed strain-specific neutralization of spirochetes in the tick midgut, albeit without killing them. Finally, we determined the ELISA serum antibody titers of infected mice against whole cell lysates of both strains (Fig 5C). Consistent with the immunoblot results, infection with either strain elicited a robust immune response, but with higher ELISA titers to homologous than heterologous strain lysates (>1x10^5^ vs 1–2.5x10^4^, respectively) (Fig 5C).

**Fig 6:**
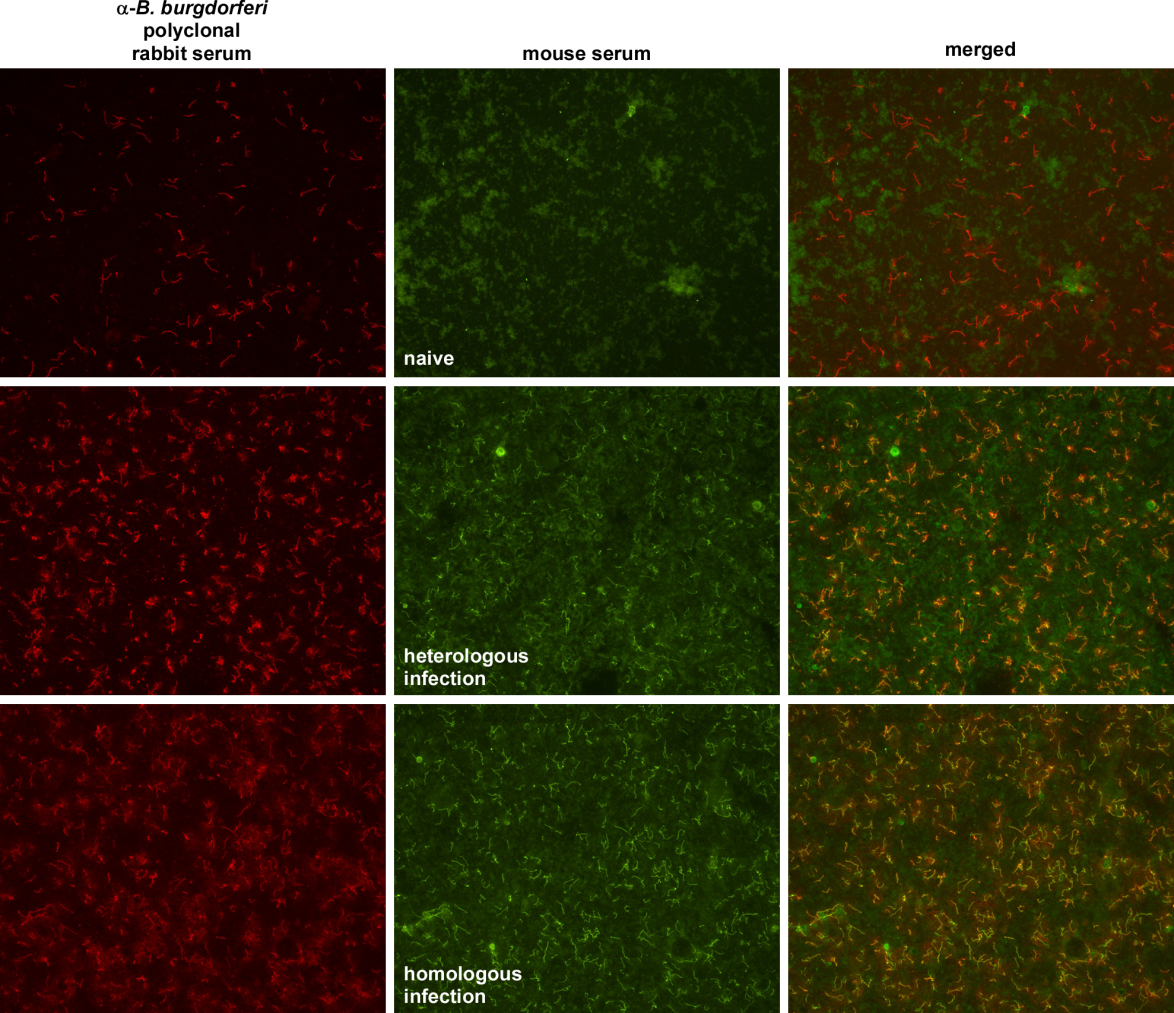
Recognition of heterologous spirochetes in infected ticks by immune mouse sera. Dissected midguts of strain B31-infected nymphs fed on naïve mice were co-stained with a rabbit polyclonal anti-*B*. *burgdorferi* serum and sera of mice infected with strain PKO (heterologous infection, middle row) or strain B31 (homologous infection, bottom row); uninfected mouse sera was used as a negative control (top row). Primary antibody binding, as identified above the panels, was visualized on a fluorescent microscope (20X magnification) with TRITC- (anti-rabbit Ig) and FITC- (anti-mouse Ig) tagged secondary antibodies, as shown in left and middle columns, respectively. Merged TRITC- and FITC images are shown in the right column.

The above analyses were conducted with whole-cell lysates of vitro grown organisms. It’s possible that cross-reactive protein antigens detected by immunoblot or ELISA analyses would not be accessible for antibody binding on intact spirochetes in the tick midgut. To address this possibility, we also compared antibody recognition of spirochetes in fed nymphs by IFA analysis, using infected mouse sera. Spirochetes in infected tick midguts stained brightly with both homologous and heterologous immune sera, whereas none were visualized by naïve mouse sera (Fig 6). This result indicates that spirochetes in the tick midgut are not “invisible” to immune recognition by the blood meal of a heterologously infected host, but that these cross-reactive antibodies do not impact the pathogenic potential of tick-borne spirochetes, whereas neutralization stems from strain-specific antibodies, such as those recognizing OspC.

### Super-infection of mice only by heterologous strain following tick challenge

The above experiments assess the pathogenic potential of spirochetes in ticks fed on naïve and infected mice, but do not evaluate the outcome for these blood-meal hosts. Previous reports indicate that super-infection by tick bite only occurs when an immune (infected) host is challenged by ticks carrying a different *B*. *burgdorferi* strain [21, 22, 26, 29, 30, 38]. We confirmed this outcome in our experimental system using unmarked strain PKo, and two infectious clones of strain B31 (S9 and A3*) that can be easily distinguished by their characteristic antibiotic resistance phenotypes. Primary mouse infections were established with either B31-S9 or PKo, as confirmed by seroconversion and isolation of spirochetes from ear punch biopsies. Infected mice were subsequently challenged by tick bite with nymphs infected with B31-A3*, and super-infection assessed several weeks later. The results were as predicted: all mice infected with strain PKo became super-infected with strain B31-A3* (10/10), whereas none of the mice infected with B31-S9 (0/20) were super-infected following a parallel challenge with B31-A3* -infected ticks. These results, coupled with our previous experiments, lead us to conclude that strain-specific antibody recognition/neutralization of spirochetes occurs within the tick midgut prior to transmission and prevents host super-infection by the same *B*. *burgdorferi* strain.

## Discussion

**Fig 7:**
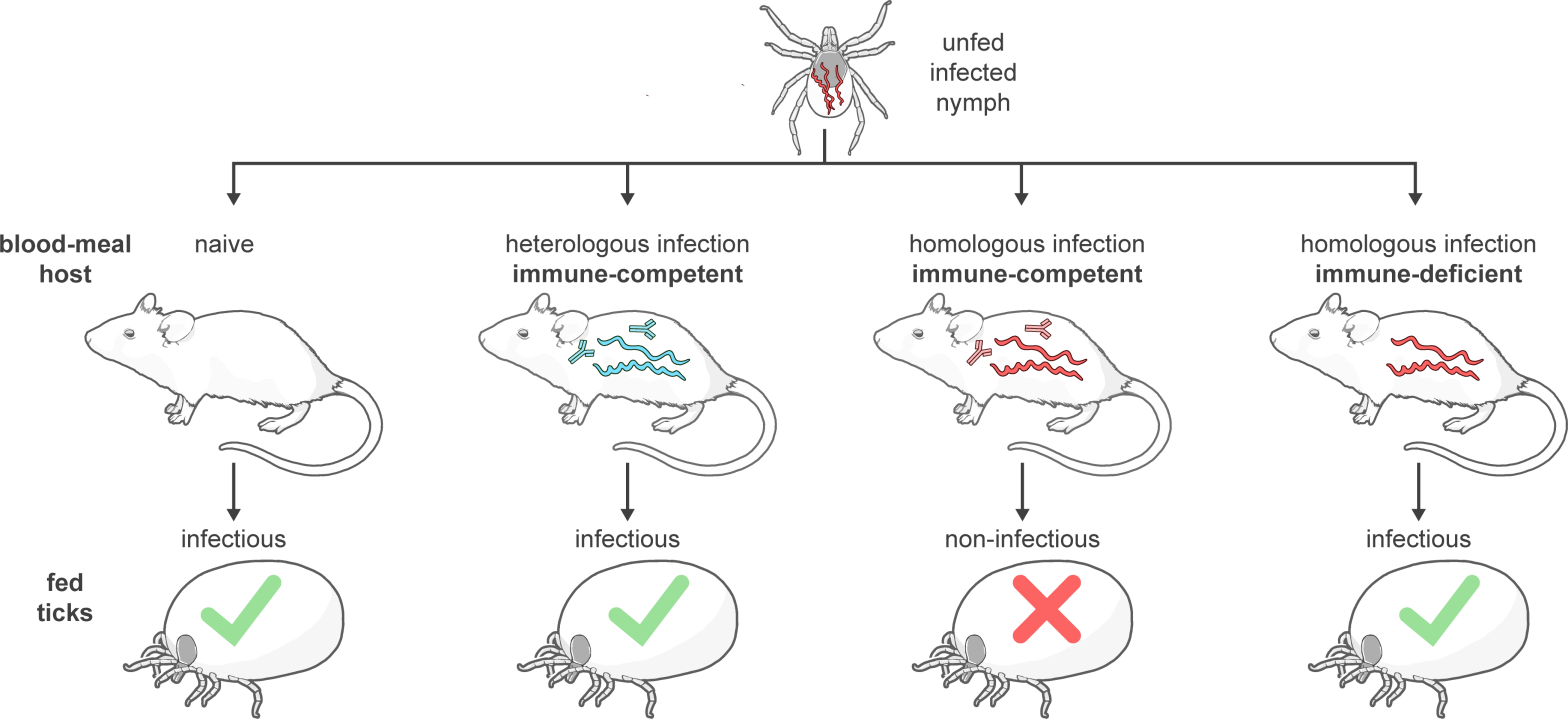
Schematic representation of the impact of host immunity on the infectivity of Lyme disease spirochetes within feeding ticks. Tick-borne spirochetes become highly infectious when the vector feeds upon an uninfected host (naïve mouse), or upon infected hosts lacking neutralizing antibodies (heterologously infected or immune-deficient). In sharp contrast, tick-borne spirochetes are non-infectious when the vector feeds upon an immune-competent host infected with the same *B*. *burgdorferi* strain (homologously infected and immune-competent). This outcome indicates that strain-specific antibodies neutralize infectious organisms within the tick midgut, prior to transmission, when the mammalian host and tick vector are infected with the same Lyme disease spirochete strain.

We and others have previously shown that activation of *B*. *burgdorferi* for vertebrate infection initiates when spirochetes in the midgut of a feeding tick encounter host blood [7–11, 32]. In the current study we demonstrate that the immune status of the host is a critical variable in the activation phenomenon we have termed “conditional priming”: blood from an infected (immune) host can also neutralize virulent spirochetes during tick feeding, but only when both vector and host are infected with the same *B*. *burgdorferi* strain, as depicted in Fig 7. In nature, multiple strains of *B*. *burgdorferi* co-exist in the same endemic area, and a high proportion of reservoir hosts are infected, sometimes with multiple strains [13, 14, 16, 18, 19]. Previous studies have demonstrated that Lyme disease patients can become re-infected by a different *B*. *burgdorferi* strain following effective antibiotic therapy [24, 30]. Our current study indicates that ingested host blood specifically targets and neutralizes (without killing) homologous spirochetes within feeding ticks, while enhancing the pathogenic potential of heterologous strains. OspC is a highly polymorphic surface protein that is recognized by strain-specific neutralizing antibodies in infected hosts [37, 39–45]. Multiple *ospC* alleles are stably maintained in natural *B*. *burgdorferi* populations, presumably through some form of balancing selection [15, 22, 43, 46–50]. The results of our current study indicate that rare *ospC* alleles would confer a fitness advantage in endemic populations, consistent with maintenance of the observed polymorphism of OspC through negative frequency-dependent selection.

We have referred to “strain-specific” neutralizing antibodies, protective immunity, etc., but the heterologous strains employed in the current study represent 2 distinct genospecies of the Lyme disease spirochete, *B*. *burgdorferi* and *B*. *afzelii*, with laboratory mice (*Mus musculus)* as the reservoir host. This experimental model resembles the enzootic cycle in Eurasia, in which several genospecies of Lyme disease spirochete co-exist and infect a variety of reservoir hosts, including wild *M*. *musculus* [18]. A single genospecies, *B*. *burgdorferi*, predominates in North America, with *Peromyscus* sp. as major reservoir hosts. However, diversity is stably maintained within local populations of *B*. *burgdorferi* in North America [14, 15, 20], consistent with immune-mediated selection of polymorphic surface antigens among spirochetes of the same genospecies.

Infected nymphs contained similar spirochete burdens after feeding on naïve or heterologously infected mice (Figs 2 and 4). This result demonstrates that spirochetes acquired during the nymphal blood meal contribute only slightly to the colonized tick midgut population. We noted a slight reduction in spirochete load when infected nymphs fed on homologously infected WT mice (Fig 4), which does not match the dramatic difference (>10,000-fold) in the infectious dose of viable spirochetes derived from these separate mouse/tick cohorts (Table 1). Theoretically, some component of the tick midgut could influence the pathogenic potential of spirochetes. However, all spirochetes compared in the current study were exposed to a similar tick midgut environment before and during feeding, irrespective of the infectious/immune status of the nymphal blood-meal host. Likewise, strain-specific neutralization of tick-derived spirochetes through in vitro exposure to immune sera (Table 4), directly demonstrates that ingested antibodies, not tick midgut components, attenuate the infectious phenotype of tick-borne spirochetes.

As an essential surface component and hallmark of host-adapted spirochetes, neutralization of OspC^+^ spirochetes by the strain-specific host immune response could explain the depletion of infectious spirochetes in fed ticks. However, OspC^+^ spirochetes were detected in the midguts of ticks fed on homologously infected (immune) hosts (Fig 3). If strain-specific antibody recognition of OspC comprises the neutralizing element of host immunity, antibody binding must somehow obscure OspC’s essential function. Alternatively, additional surface components of *B*. *burgdorferi* could be targets of neutralizing immunity, as infected mouse sera recognize a number of proteins in a strain-specific fashion (Fig 5).

It is puzzling that genetically identical, wild-type spirochetes in the midgut of an infected tick do not uniformly undergo a host-adaptive response with respect to OspC induction during tick feeding (Figs 3 and S3) [51–53]. This could reflect exposure of individual spirochetes to different cues in the local tick midgut micro-environment, but spirochetes that lack OspC are not infectious, even when successfully transmitted to a naïve host [32, 33, 51]. Perhaps this seemingly imperfect genetic program confers a fitness advantage under negative frequency-dependent selection, as our current study demonstrates that host-adapted, infectious spirochetes are selectively neutralized when ticks ingest blood from an immune host carrying the same strain (*ospC* allele).

A previously marketed Lyme disease vaccine for humans (Lymerix) [54] targeted OspA, which is a relatively well-conserved surface component of spirochetes colonizing the tick midgut [7, 52, 55, 56]. Vaccination with OspA elicits antibodies that recognize spirochetes in the tick midgut and prevent transmission [57–60]. Although OspA represents an effective vaccine target, conditional priming of spirochetes during tick feeding results in down-regulation of OspA and induction of OspC, as required for host infection and subsequent larval acquisition [32, 52]. Consistent with this scenario, *B*. *burgdorferi*-infected mice do not make antibodies against OspA, and hence OspA is not a target of strain-specific neutralizing immunity in the current study.

OspC has also received attention as a vaccine candidate because it is an indispensable and abundant surface component of the Lyme disease spirochete that naturally elicits a strong neutralizing immune response during host infection [42, 61–66]. However, the high degree of OspC polymorphism among *B*. *burgdorferi* strains in local endemic regions, and resulting strain-specific immune response, present a substantial challenge to an OspC-based vaccine [15, 40, 67, 68]. These challenges were addressed by Earnhart and colleagues with a multivalent chimeric Lyme disease vaccine that incorporates neutralizing linear epitopes from multiple OspC types [69]. These investigators have recently proposed to include a linear epitope of OspA that elicits bactericidal antibodies, but does not encompass a putative autoimmune epitope [70]. A vaccine targeting both OspA and OspC would theoretically neutralize both host-adapted (OspA-, OspC+) and uncommitted (OspA+, OspC-) spirochetes in the tick midgut, prior to transmission to a mammalian host.

It is well established that the Lyme disease spirochete must undergo an adaptive response during tick feeding in order to infect the vertebrate host [6–11]. We previously found that transmission of *B*. *burgdorferi* during the nymphal blood meal represents a bottleneck through which only a random subset of infectious tick-borne spirochetes can pass and successfully infect naïve hosts [71]. In the current study we demonstrate that the immune response of an infected vertebrate host specifically ablates the infectious phenotype of homologous spirochetes within feeding ticks, prior to transmission and without killing them. These findings provide insight into the evolutionary processes that shape the natural diversity of the Lyme disease spirochete and expose a point in the transmission cycle that is inherently restricted and highly vulnerable to the vertebrate immune response.

## Materials and methods

### Ethics statement

The Rocky Mountain Laboratories, National Institute of Allergy and Infectious Diseases, National Institutes of Health, Animal Care and Use Committee (RML, NIAID, NIH, IACUC; USDA Permit Number: 51-F-0016 Customer #441, PHS number: A-4149-01) approved study protocols for work conducted in strict accordance with the Guide for the Care and Use of Laboratory Animals of the National Institutes of Health. All infection studies were performed in an Animal Biosafety Level 2 facility according to protocols reviewed and approved by the RML Institutional Biosafety Committee and the RML IACUC. All work in this study adhered to the institution’s guidelines for animal husbandry, and followed the guidelines and basic principles of the Public Health Service Policy on Humane Care and Use of Laboratory Animals. Mice were anesthetized by isoflurane inhalation prior to inoculation or blood withdrawal. Mice were sedated with a ketamine/xylazine cocktail for tick feeding experiments. Mice were euthanized by isoflurane inhalation followed by cervical dislocation.

### Bacterial strains and culture conditions

Strains B31-A3-68Δe02 (S9) and B31-A3-lp25Gm (A3*) are infectious clonal derivatives of *B*. *burgdorferi* sensu strictu type strain B31 (ATCC35210) [1, 72, 73]. Strains B31-S9 and B31-A3* carry antibiotic resistance cassettes that permit growth in streptomycin or gentamicin, respectively, and differ in the presence (A3*) or absence (S9) of endogenous restriction/modification systems [73]. Wild-type (WT) strain PKo is an infectious *B*. *afzelii* strain originally isolated from human skin [74]. Spontaneous B31 and PKo variants that no longer make OspC were used as control lysates on some immunoblots. Liquid cultures were inoculated from frozen stocks and propagated with minimal passage in Barbour-Stoenner-Kelly II (BSK II) medium containing gelatin and 6% rabbit serum, and supplemented with 50μg/ml streptomycin (S9) or 40μg/ml gentamicin (A3*), where appropriate. Viable spirochetes were quantified as colony forming units (CFUs) in solid BSK medium incubated at 35°C with 2.5% CO_2_ [75].

### *B*. *burgdorferi* s.l. infection in mice

Immune-competent RML mice are derived from a colony of Swiss-Webster mice established at NIH in 1935 and maintained at the Rocky Mountain Laboratories with an outbreeding program designed to intentionally maintain genetic diversity. RML mice reject autologous grafts, demonstrating MHC diversity within the colony. Immune-competent C57BL/6J ("B6") and immune-deficient B6.129S7-Rag1^tm1Mom^/J ("Rag1 KO") and B6.129S2-Ighm^tm1Cgn^/J (“muMT^-^) inbred mice were obtained from Jackson Laboratories, Bar Harbor, ME. C57Bl/6 is a non-albino inbred mouse strain that is not derived from Swiss-Webster mice. Mice (six to eight weeks old) used in tick feeding experiments were infected by needle inoculation with a dose of approximately 10^4^ spirochetes (8×10^3^ intraperitoneally and 2×10^3^ subcutaneously). Spirochetes were enumerated using a Petroff-Hausser chamber and diluted in BSK II for the inocula. Mice were bled three weeks after injection and assessed for seroconversion, as described below. All experiments assessing the infectivity of spirochetes in tick homogenates by needle inoculation of naïve mice, as described below, were conducted with homogenates prepared from B31-S9-infected nymphs fed upon naïve, PKo-infected (heterologous) or B31-S9-infected (homologous) mice. The subset of experiments addressing mouse super-infection by tick-bite challenge was conducted with B31-A3*-infected nymphs fed upon PKo-infected (heterologous) or B31-S9-infected (homologous) mice.

### Immunoblots

Whole-cell lysates of B31-S9 were separated by electrophoresis through 12% polyacrylamide gels and transferred to nitrocellulose membranes. Blots were blocked with 5% nonfat milk in TBST (Tris-buffered saline, 0.1% Tween 20) for 1 h at room temperature, followed by incubation with mouse sera (1:200 dilution in TBST) at 4°C overnight. Blots were washed 3 times with TBST, 15 minutes per wash, and then incubated for 1–2 h with peroxidase-conjugated sheep anti-mouse IgG or goat anti-mouse polyvalent immunoglobulins (1:10,000 dilution in TBST) (Sigma-Aldrich, St Louis, MO). Blots were again washed 3 times in TBST before incubation with enhanced chemiluminescent reagents (SuperSignal, Pierce Thermo Scientific, Rockford, IL) and exposure to X-ray film. Multiple proteins were recognized by sera of infected mice, while no bands were present on immunoblots using sera from pre-immune or uninfected mice. A typical immunoblot with infected (seropositive) and uninfected (seronegative) sera is shown in S1 Fig.

### ELISA

The relative serum antibody titers of infected WT mice against homolgous or heterologous strains were determined by enzyme-linked immunosorbent assay (ELISA). 5 mice per group were injected with ~10^4^ strain B31-S9 or strain PKo spirochetes. Pre-immune sera was obtained from all mice prior to injection and infected sera obtained 3 weeks after inoculation. Pooled sera from all 5 mice in each group was used to determine antibody titers before and after infection. Stationary phase cultures of B31-S9 and PKo were harvested by centrifugation, washed twice in phosphate-buffered saline (PBS; pH 7.4) and resuspended in PBS at 1/10^th^ of the original culture volume. Whole cell lysates of spirochetes were prepared by sonication of PBS suspension on ice using a Heat Systems Ultrasonic Processor (XL-2015) sonicator (Misonix, Farmingdale, NY) at 40% amplitude with four repetitions of 20 s each. The total protein concentration of B31-S9 and PKo lysates was estimated using Bradford reagent (Sigma-Aldrich) and normalized to 1 mg/ml with PBS. Immulon 2 HB 96-Well ELISA plates (Thermo Fisher Scientific, Bothell, WA) were incubated overnight at 4°C with 1 μg of B31-S9 or PKo lysate per well in coating buffer (0.1 M carbonate-bicarbonate buffer, pH 9.6). Non-specific binding sites were blocked with 5% non-fat dry milk in PBS containing 0.2% Tween-20 (PBST). Serial dilutions of each pooled sera (100 μl/well) were added to triplicate wells in blocking buffer and incubated for 2 h at 37°C. HRP-conjugated rabbit anti-mouse IgG was then added to each well (1:10,000) and incubated at 37°C for 1 h. Wells were washed several times with PBST and color was developed using ABTS 2-Component Microwell Peroxidase Substrate Kit (SeraCare, Milford, MA). The reaction was terminated by the addition of 1% SDS in water and absorbance measured at 405 nm with an ELISA plate-reader (Labsystems Multiskan Plus, Thermo Fisher Scientific). Baseline absorbances were determined for each dilution of pooled pre-immune sera, in triplicate, against B31-S9 and PKo lysates. The threshold for positive sero-reactivity was set at 3 standard deviations above the mean absorbance of pre-immune serum at each dilution. The titer of infected mouse sera against heterologous and homologous strains represents the highest dilution at which absorbance above this baseline cut-off was achieved.

### Larval tick feeding and *B*. *burgdorferi* acquisition to generate infected nymphs

Infected RML mice were infested with 100–150 larval *Ixodes scapularis* ticks (Oklahoma State University). Fully engorged larvae were collected daily as they dropped off the host. Eight to ten days later, several fed larvae per mouse were mechanically disrupted as described below, and cultured in BSKII medium to confirm colonization of 80–100% of ticks. The remainder of each infected larval cohort was allowed to molt into nymphs.

### Preparation of homogenates from fed infected nymphs and quantification of viable spirochetes

Cohorts of strain B31-infected nymphs were allowed to feed to repletion on naïve or infected mice (~20 nymphs/mouse) and collected at drop-off. Several ticks from each mouse were used in IFA analyses, as described below and shown in Figs 3 and S3. The remaining fed nymphs for each mouse were pooled, surface-sterilized by sequential immersion for 5 minutes in 3% hydrogen peroxide and 70% ethanol, and homogenized in BSK medium in sterile 1.5 ml microfuge tubes with disposable plastic pestles (Kimble Chase, Rockwood, TN). Aliquots of pooled tick homogenates for each mouse were plated to enumerate viable bacteria as CFU and the remainder frozen at -80°C. The average spirochete burden per tick for each pool of ticks fed on an individual mouse was estimated, as shown in Figs 2 and 4. Enumeration of viable spirochetes as CFU was repeated when tick homogenates were thawed, serially diluted, and inoculated into naive RML mice, as described below.

### Assessment of infectious phenotype

The relative infectious dose or pathogenic potential of spirochetes in tick homogenates was assessed in several independent experiments. In each experiment, groups of 3 to 6 naïve wild-type RML mice received 10-fold-increasing doses of tick-derived spirochetes, ranging from approximately 10 to 10^5^ organisms per mouse. Mice were bled and euthanized 4 weeks after inoculation, and ear, bladder, and ankle joint tissues were harvested and incubated in BSKII medium. Mouse infection was determined by seroconversion and isolation of spirochetes from tissues (Tables 1–4, S1). A representative immunoblot with positive and negative sera is shown in S1 Fig.

### Exposure of infected tick homogenates to mouse sera

Aliquots of a highly infectious homogenate prepared from B31-infected nymphs fed upon naive mice (infectious dose ~10 spirochetes, Table 1) were exposed to equal volumes of sera from naive or infected mice for 30 minutes at room temperature. An aliquot of the same infected tick homogenate was incubated with PBS as an untreated control. 10^3^ organisms of each treated homogenate were injected into naïve mice to assess the impact of serum exposure on spirochete infectivity, as described above. A dilution of each inoculum was also plated to confirm the viability of spirochetes after incubation with serum or PBS.

### Immunofluorescence assay

Spirochetes in dissected tick midguts were visualized by immunofluorescence assay (IFA) on a fluorescent microscope (20X magnification) with a polyclonal rabbit anti-*B*. *burgdorferi* primary antiserum and a TRITC-labeled secondary antibody (Kirkegaard & Perry Laboratories, Gaithersburg, MD), while synthesis of OspC by these spirochetes was examined using a mouse monoclonal anti-OspC primary antibody {provided by Robert Gilmore; [61]} and a FITC-labeled secondary antibody (Kirkegaard & Perry Laboratories, Gaithersburg, MD). Specificity of antibody staining was confirmed using uninfected tick midguts or by omission of primary or secondary antibodies (S2 Fig) as negative controls.

### Statistical analyses

Differences in the outcome of infection in mice (Tables 1, 2, 3, 4 and S1) were analyzed by Fisher exact test and P values < 0.05 were considered significant. Spirochete burdens in ticks (Figs 2 and 4) were analyzed using the GraphPad software PRISM 7 and P values were calculated using non-parametric rank order test.

## Supporting information

S1 Fig Representative immunoblots of seropositive and seronegative mouse sera. Mice inoculated with homogenates of strain B31-infected ticks fed upon naïve mice (lanes 1–3) were seropositive, as were mice inoculated with homogenates of B31-infected ticks fed upon strain PKo-infected mice (heterologously infected, lanes 7–9), whereas mice inoculated with homogenates of B31-infected ticks fed upon strain B31-infected mice (homologously infected, lanes 4–6) were seronegative. Separate blot strips were used with each serum sample and roughly aligned for exposure to X-ray film. Antibody binding was visualized by incubation of the blots with a peroxidase-labeled secondary anti-mouse IgG antibody and chemi-luminescent reagents.
(TIFF)

S2 Fig Control IFA images demonstrating specificity of antibody staining. Dissected midguts of strain B31-infected fed nymphs were incubated with primary or secondary antibodies alone, as identified beneath the images, to control for background autofluoresence of the tick mid-gut (left panel) and non-specific binding of fluorescently-tagged secondary antibodies (middle panel). The dissected midgut of an uninfected fed nymph was incubated with both primary and secondary antibodies (right panel) to demonstrate specificity of antibody staining. Merged TRITC and FITC images are shown for all.
(TIF)

S3 Fig OspC production by spirochetes in infected nymphs fed on naïve or infected muMT^-^ mice. Dissected midguts of strain B31-infected nymphs fed on naïve or infected muMT^-^ mice, as identified to the right of the images, were co-stained with a rabbit anti-*B*. *burgdorferi* polyclonal serum and a mouse monoclonal antibody that selectively stains spirochetes synthesizing OspC. Primary antibody binding, as identified above the panels, was visualized on a fluorescent microscope (20X magnification) with TRITC- (total *B*. *burgdorferi*) and FITC- (OspC+ *B*. *burgdorferi*) tagged secondary antibodies. The presence of midgut spirochetes making OspC was confirmed by visual assessment of IFA slides from 6 nymphs per experimental group, and 5 fields per nymph. Similar IFA results were obtained with B31-infected nymphs fed on naïve or infected Rag1 KO mice.
(TIF)

S1 Table Infectivity of spirochetes in infected nymphs after feeding on naïve or infected wild-type C57Bl/6 mice. (DOCX)
