# Supplementary figures and images for: Infection history of the blood-meal host dictates pathogenic potential of the Lyme disease spirochete within the feeding tick vector
Source: PLoS Pathog. 2018 Apr 5;14(4):e1006959. doi: 10.1371/journal.ppat.1006959 (PMC5886588; doi:10.1371/journal.ppat.1006959)

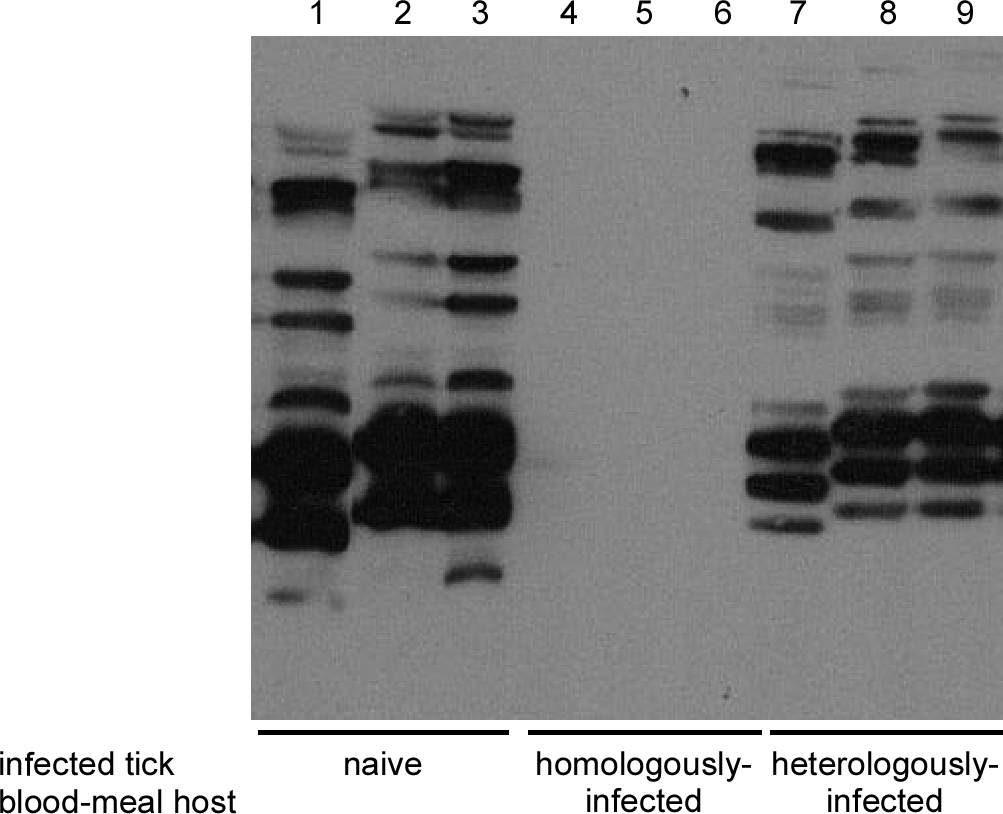

Supplement: S1 Fig — Mice inoculated with homogenates of strain B31-infected ticks fed upon naïve mice (lanes 1–3) were seropositive, as were mice inoculated with homogenates of B31-infected ticks fed upon strain PKo-infected mice (heterologously infected, lanes 7–9), whereas mice inoculated with homogenates of B31-infected ticks fed upon strain B31-infected mice (homologously infected, lanes 4–6) were seronegative. Separate blot strips were used with each serum sample and roughly aligned for exposure to X-ray film. Antibody binding was visualized by incubation of the blots with a peroxidase-labeled secondary anti-mouse IgG antibody and chemi-luminescent reagents. (TIFF) [file ppat.1006959.s001.tiff]

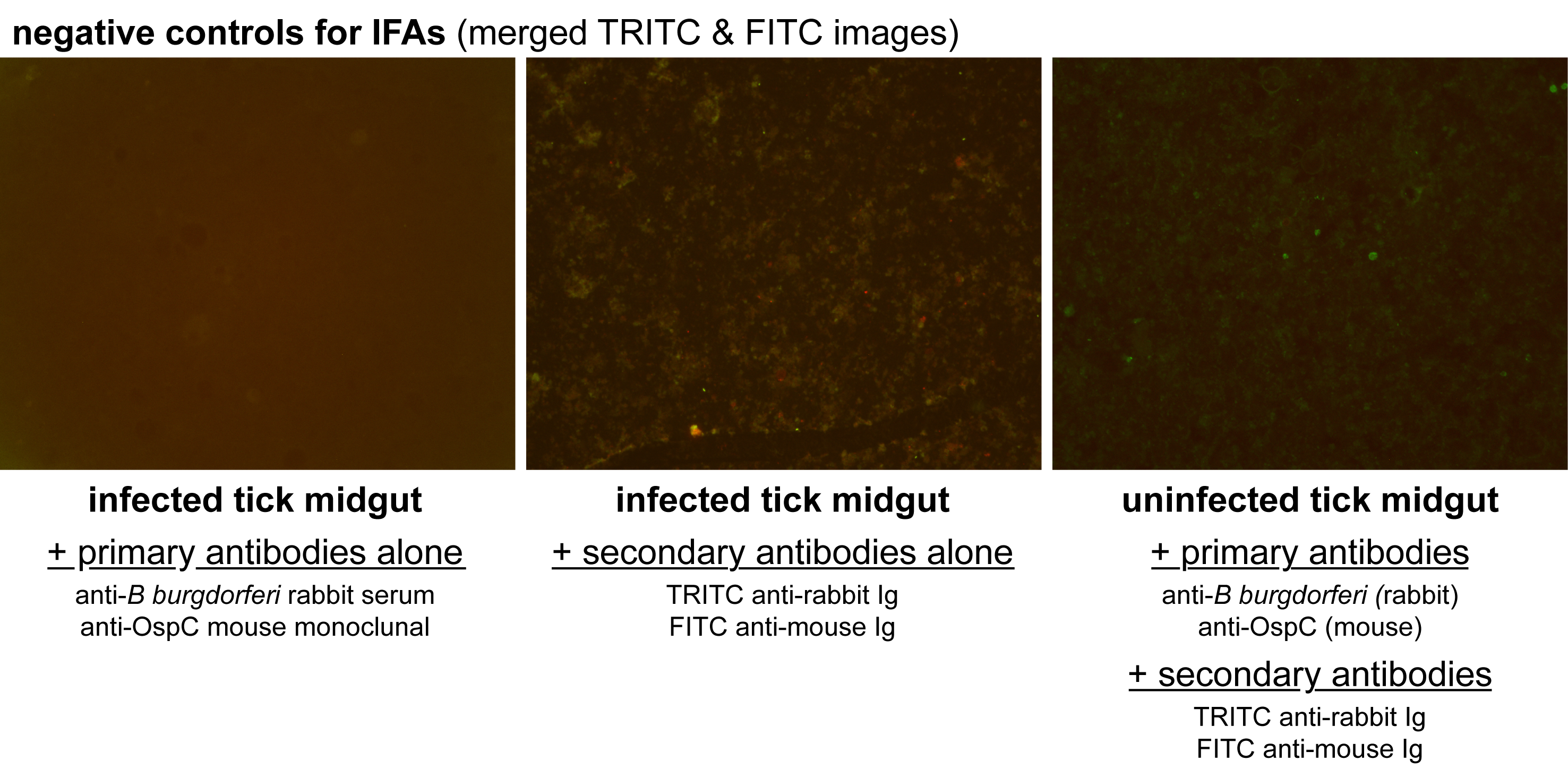

Supplement: S2 Fig — Dissected midguts of strain B31-infected fed nymphs were incubated with primary or secondary antibodies alone, as identified beneath the images, to control for background autofluoresence of the tick mid-gut (left panel) and non-specific binding of fluorescently-tagged secondary antibodies (middle panel). The dissected midgut of an uninfected fed nymph was incubated with both primary and secondary antibodies (right panel) to demonstrate specificity of antibody staining. Merged TRITC and FITC images are shown for all. (TIF) [file ppat.1006959.s002.tif]

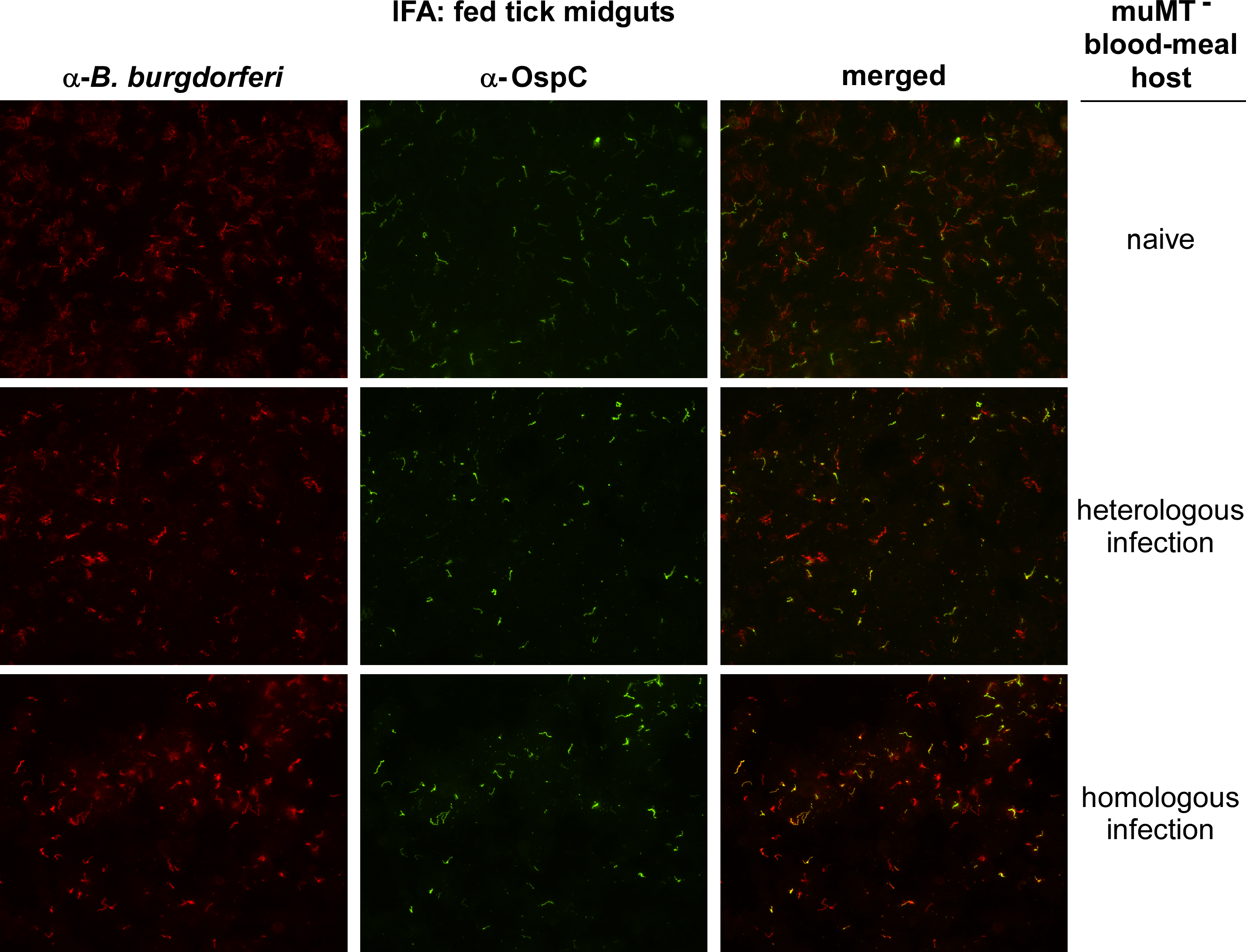

Supplement: S3 Fig — Dissected midguts of strain B31-infected nymphs fed on naïve or infected muMT- mice, as identified to the right of the images, were co-stained with a rabbit anti-B. burgdorferi polyclonal serum and a mouse monoclonal antibody that selectively stains spirochetes synthesizing OspC. Primary antibody binding, as identified above the panels, was visualized on a fluorescent microscope (20X magnification) with TRITC- (total B. burgdorferi) and FITC- (OspC+ B. burgdorferi) tagged secondary antibodies. The presence of midgut spirochetes making OspC was confirmed by visual assessment of IFA slides from 6 nymphs per experimental group, and 5 fields per nymph. Similar IFA results were obtained with B31-infected nymphs fed on naïve or infected Rag1 KO mice. (TIF) [file ppat.1006959.s003.tif]
