# Supplementary material for: Infection history of the blood-meal host dictates pathogenic potential of the Lyme disease spirochete within the feeding tick vector
Source: PLoS Pathog. 2018 Apr 5;14(4):e1006959. doi: 10.1371/journal.ppat.1006959 (PMC5886588; doi:10.1371/journal.ppat.1006959)
Supplement: S1 Table — (DOCX) [file ppat.1006959.s004.docx]

| **infection status of blood-meal host^1^** | **number of spirochetes in inoculum^2^** | **number of mice infected/total naïve mice injected^3^** | |
| --- | --- | --- | --- |
| **naive** | 10  10^2^  10^3^  10^4^ | 4/5  4/5  5/5  3/3 | **16/18** |
| **heterologous** | 10  10^2^  10^3^  10^4^ | 3/5  5/5  5/5  3/3 | **16/18** |
| **homologous** | 10  10^2^  10^3^  10^4^ | 0/5  0/5  1/5  0/3 | **1/18*** |

^1^ Cohorts of nymphs infected as larvae with *B. burgdorferi* strain B31 were fed to repletion on groups of naïve, heterologously-infected (PKo) or homologously-infected (B31) wild-type C57Bl/6J mice (blood-meal host).

**Table S1. Infectivity of tick-borne spirochetes after feeding on naïve or infected wild-type C57Bl/6J mice.**

^2^ Homogenates prepared from pools of fed nymphs were used to needle-inoculate naïve wild-type mice with doses ranging from 10-10^4^ viable spirochetes per mouse, as enumerated and confirmed by plating.

^3^ *B. burgdorferi* infection in mice was determined by seroconversion to whole cell lysates by immunoblot analysis, and isolation from ear, bladder and joint tissues; infected mice were positive by all measures. *P = 6.45e-7 for homologous versus naïve; P = 6.45e-7 for homologous versus heterologous; P = 1 for heterologous versus naive blood-meal hosts. P values calculated using Fisher’s exact test for infectious outcomes with different blood-meal hosts.
